# Insights into the Mechanism of Severe Mitral Regurgitation: RT-3D TEE Guided Management with Pathological Correlation

**DOI:** 10.1155/2015/961565

**Published:** 2015-11-12

**Authors:** Senthil Anand, Naktal Hamoud, Jess Thompson, Rajesh Janardhanan

**Affiliations:** ^1^Department of Medicine, Banner-University Medical Center South, Tucson, AZ 85713, USA; ^2^Department of Cardiovascular Medicine, Sarver Heart Center, Banner-University Medical Center, Tucson, AZ 85724, USA

## Abstract

Mitral valve perforation is an uncommon but important complication of infective endocarditis. We report a case of a 65-year-old man who was diagnosed to have infective endocarditis of his mitral valve. Through the course of his admission he had a rapid development of hemodynamic instability and pulmonary edema secondary to acutely worsening mitral regurgitation. While the TEE demonstrated an increase in the size of his bacterial vegetation, Real Time 3D TEE was ultimately the imaging modality through which the valve perforation was identified. Through this case report we discuss the advantages that RT-3D TEE has over traditional 2D TEE in the management of valve perforation.

## 1. Case Presentation

**Figure 1:**
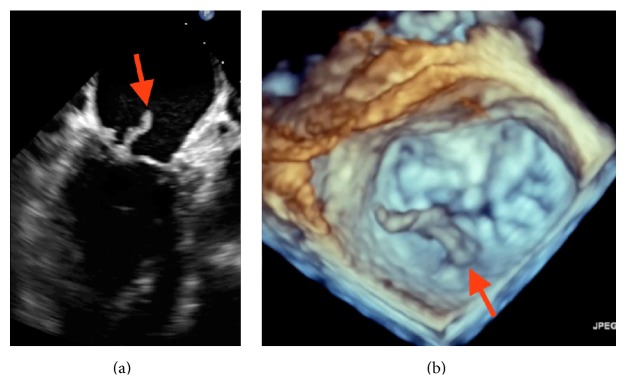
(a) shows the large vegetation on the P2 scallop of the posterior mitral valve on 2D TEE. (b) Corresponding mass noted on RT-3DTEE.

**Figure 2:**
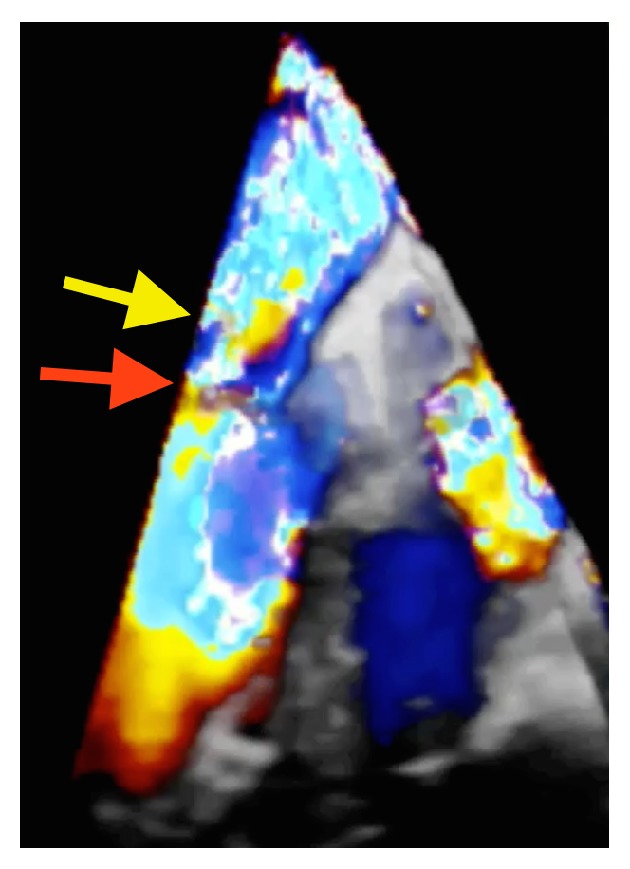
Color 3D TEE image on an Apical 4 Chamber view shows the impressive lateral jet (red arrow) originating from the perforation on the P2 scallop of posterior mitral leaflet. The less impressive central jet of mitral regurgitation is partially seen in this image (yellow arrow).

**Figure 3:**
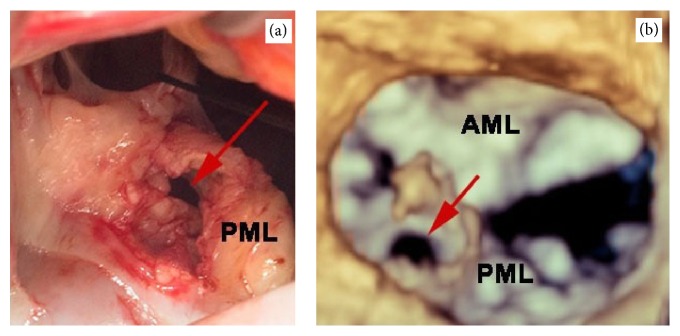
The red arrows on RT-3DTEE (a) and intraoperative image of the mitral valve (b) showing the perforation in the P2 scallop of the posterior mitral leaflet (AML: anterior mitral leaflet; PML: posterior mitral leaflet).

A 65-year-old man was admitted with severe sepsis and bacteremia. He endorsed malaise, myalgia, fever, chills, and reduced appetite. His blood cultures were positive for Methicillin-sensitive* Staphylococcus aureus*. On admission, he was found to have acute renal failure and multiple septic brain emboli. The initial transthoracic echocardiogram (TTE) showed mild mitral regurgitation (MR) with no vegetation. In the presence of positive blood cultures and recurrent septic emboli, a 2D-transesophageal echocardiogram (TEE) was done on day 4 that revealed a 1.3 cm × 0.3 cm wide mobile mass on the atrial side of the posterior mitral leaflet, suggestive of vegetation. While the patient was getting appropriate antibiotics, on day 12, he developed an acute pulmonary edema and new onset of atrial fibrillation with rapid ventricular rate. 2D-transesophageal echo revealed a much larger vegetation (Figure 1(a)) measuring 2.3 cm on the P2 scallop of the posterior leaflet of the mitral valve and severe MR. Real Time 3D Transesophageal Echocardiography (RT-3D TEE) confirmed a large vegetation on the P2 scallop of the posterior mitral leaflet (Figure 1(b)). Color 3D Doppler revealed two mitral regurgitation jets in the left atrium, one jet with a central origin and one jet that was lateral (Figure 2), and we were able to easily identify a perforation in the P2 scallop of the posterior mitral leaflet with an estimated area of 0.8 cm. The surgery team was consulted and on day 25 the patient underwent a repair of the mitral valve with placement of a 25 mm Simulus flexible annuloplasty band. Intraoperatively, the perforation size and location on the P2 scallop of the posterior mitral valve (Figure 3(a)) exactly matched the preoperative 3D TEE findings (Figure 3(b)).

## 2. Discussion

Despite appropriate medical management of our patient's infective endocarditis there was a rapid development of hemodynamic instability resulting in pulmonary edema. This raised the suspicion that the patient had developed a structural valvular complication secondary to the infective endocarditis. RT-3D TEE confirmed valve leaflet perforation and thus provided a mechanistic insight into the new onset severe MR. The ability to manipulate the images in a three-dimensional environment also enabled us to better communicate the valve pathology to the surgical team and guide further treatment including valve repair.

Mitral valve perforation is an uncommon but important complication of bacterial endocarditis [1]. While TEE is much superior to TTE in the diagnosis of mitral leaflet perforation (sensitivity 95%, specificity 98) [2], RT-3D TEE has been shown to provide additional qualitative information on the mitral valve apparatus when compared with 2D TEE [3]. With its ability to attain en face anatomic views of the cardiac valves, RT-3D TEE has been shown to have higher sensitivity at detecting valve perforations, as well as better ability to characterize their size and location when compared with traditional 2D TEE [4]. RT-3D TEE provides optimal identification of the morphology and pathology of the mitral valve apparatus [5], and in endocarditis it has been reported to have high sensitivity and specificity [6].

In conclusion, this case demonstrates the important diagnostic value of RT-3DTEE for mitral valve perforation. Without any additional risk to the patients, the use of RT-3D can improve the diagnostic power of TEE for valvular perforation; also, it can clearly identify the site of the perforation with accurate estimate of the size of the perforation and provides surgeons with relevant details to plan valve repair. Excellent surgical results have been reported after 3D TEE in such patients. We suggest that 3D techniques should be part of the standard TEE in all patients with endocarditis to ensure high diagnostic accuracy and anatomical information.
